# 25, 50 & 75 years ago

**DOI:** 10.1111/ans.18061

**Published:** 2022-10-11

**Authors:** Julian A. Smith

**Affiliations:** ^1^ Department of Surgery Monash University Melbourne Victoria Australia

## Twenty‐five years ago

**Gordon NSI, Hadlow G, Knight E, Mohan P. Transurethral resection of the prostate: still the gold standard. *ANZ. J. Surg*. 1997; 67: 354–7.**

In recent times, there has been a number of newer methods advocated as treatment for bladder outlet obstruction. Prior to embracing these newer technologies, the authors' experiences with conventional transurethral resection of the prostate (TRUP) should be evaluated and compared with those experienced in the newer modalities. The objective was to determine whether a standard TURP still compared favourably with the newer modalities in terms of duration of stay, duration of catheterization, re‐admission rate, re‐catheterization rate, cost and long‐term results. The results are compared with those of workers whose level of expertise was the best that could be achieved with transurethral needle ablation (TUNA) and laser prostatectomy. During the 3‐year period from September 1992 to September 1995, 575 TURP were carried out in a regional hospital. The total duration of stay, the postoperative duration of stay, the re‐catheterization and re‐admission rates were assessed and the costs estimated. TURP was shown to compare favourably in terms of the duration of hospital admission and the duration of catheterization, and to have a significantly lower re‐catheterization rate and a significantly lower re‐admission rate than the newer modalities. TURP is still the method of choice for surgical management of bladder outlet obstruction, and it remains as the gold standard. Having reviewed the results of the newer modalities as carried out by the experts in those fields, it was found that TURP compares favourably with those procedures. From the point of view of duration of stay, duration of catheterization, re‐admission rate and re‐catheterization rate, as well as cost and long‐term results, TURP remains as the gold standard and the newer modalities are not believed to be advantageous at this stage.

**Morgan G, Leong T, Berg D. Management of seminoma of the testis: recommendations based upon treatment results. *ANZ. J. Surg*. 1997; 67: 15–20.**

The results of management of seminoma of the testis at the Department of Radiation Oncology, St Vincent's Hospital, Sydney was evaluated retrospectively to: (i) establish that outcomes were in keeping with published results from centres in Australia and overseas; (ii) assess the impact of chemotherapy on management; and (iii) to determine ‘best practice· management protocols based on our results and a review of the relevant literature. Methods included (i) assessment of treatment results for Stage I and II seminoma of the testis treated by post‐orchidectomy radiotherapy and/or chemotherapy at St Vincent's Hospital between 1979 and 1993; (ii) literature review of published data from Australian and overseas centres on the management of seminoma of the testis, and in particular the use of surveillance or chemotherapy either alone, at time of relapse or combined with radiotherapy; and (iii) development of recommendations for use as management protocols in our department. Our data and a review of the literature suggest that post‐orchidectomy radiotherapy with chemotherapy for relapse in Stage I and IIA disease results in long‐term cure rates approaching 100%. Treatment with chemotherapy either routinely or selectively or using a surveillance policy is unlikely to show any improvement in outcome and may be less cost‐effective and/or produce increased morbidity and the risk of secondary leukaemia. For stage IIB disease (5–10 cm) the use of initial combination chemotherapy with or without subsequent radiotherapy did not appear to give better outcomes than initial radical radiotherapy alone, reserving chemotherapy or further radiotherapy for relapse. For bulkier stage IIB disease (>10 cm), the use of initial chemotherapy plus consolidation radiotherapy appeared to be an appropriate treatment. Management protocols for seminoma of the testis at St Vincent's Hospital Sydney Department of Radiation Oncology currently are (i) Stage I, IIA, and IIB (5–10 cm): post‐orchidectomy radiotherapy alone with chemotherapy or further radiotherapy for relapse; and (ii) stage IIB (>10 cm) disease: initial chemotherapy post‐orchidectomy followed by radiotherapy to sites of initial disease involvement.

## Fifty years ago

**McKenzie AR. Congenital dislocation of the hip: a 12‐year survey. *ANZ. J. Surg*. 1972; 41: 219–26.**

A 12‐year review has been made of 114 cases of congenital dislocation of the hip joint in children treated in one institution. The general plan of treatment for all cases was similar and patterned on the practice of Scott and Somerville of Oxford. Ninety‐seven hip joints in 61 patients were re‐examined clinically and radiologically, and the results are analysed.

The review records an overall proportion of 60% of satisfactory and good hips in children diagnosed as ‘congenital dislocation of the hip joint’ at a late age. There are no new or original observations to record. Clinical assessment does not closely correspond with radiological assessment. This is in accord with the findings of Smith *et al*. (1968). A good skiagram is more likely to be associated with a good clinical result, and a large number of the patients with unsatisfactory clinical results will have unsatisfactory skiagrams.

Better overall results in the future will be achieved by earlier diagnosis and the observance of extreme gentleness when dealing with the hip joints of young children. Such gentleness of handling would seem to be of little value if the young hip joint is held in an extreme posture which is likely to ‘wind up’ the capsule, and so constrict or occlude the vascular channels to and from the femoral head epiphysis, and perhaps also the rim of the acetabulum. The Oxford frame, despite its gentle progressive reduction by traction and abduction, would seem to hold the hip in an extreme extended position, which is thought to be a cause of vascular insufficiency. This, at least, would seem to be the case in the experimental animal (Salter, 1967). Such an extreme position is the chief objection to the ‘frog’ position. The modified gallows and the Alvie type of traction‐abduction seem attractive alternatives. Even when avoiding such extreme postures but still keeping the hip joint immobile for long periods, it would seem to be a very likely cause of articular problems in the treated hips. Joint movements probably ensure adequate cartilage nutrition (Salter and Field, 1960; Maroudas *et al*. 1968). There seems to be, therefore, a greater place for the use of abduction splints or plasters which leave the hips free to move and flex as soon as possible after the surgery used to stabilize the reduction achieved by closed methods.

Somerville (1967) has pointed out that a hip can function remarkably well for many years in childhood, even though it is anatomically thoroughly unsatisfactory. Taken a stage further, the conclusions of Smith *et al*. (1968) of Ann Arbor are relevant, that a disturbing number of hips with theoretically perfect reductions wear out or work their way out if the observations on them are carried out for a long enough period.

**Rush JH. The surgery of acromio‐clvicular joint dislocation. *ANZ. J. Surg*. 1972; 42: 38–41.**

A classification of acromio‐clavicular joint injuries is presented, and the treatment of each type is discussed. Twenty‐three patients with complete acromio‐clavicular joint dislocation treated by surgery at the Royal Melbourne Hospital are reviewed. This small review highlights the problems associated with internal fixation of a complete dislocation of the acromio‐clavicular joint. The trans‐articular Kirschner wire technique is associated with a very high failure rate. This figure could probably be improved by attention to surgical details, such as meticulous reduction of the joint with removal of any obstructing tissue such as capsule or intra‐articular disc. The wire must be well placed in both acromion and clavicle, and the superior acromio‐clavicular ligament should be repaired. Although migration of the wire into the axilla is often quoted as a complication, this did not occur in this series. In addition, there was no case in which the wires broke and re‐dislocation occurred. The follow‐up of these cases is too short for investigation of the problem of degenerative arthritis, which is said to occur if the trans articular Kirschner wire technique is used (Jacobs and Wade, 1966; Quigley, 1963). The other major surgical alternative is to use a lag screw inserted across the clavicle and into the coracoid. The screw must be removed at 12 weeks, when the coracoclavicular ligament will have healed. If this is not done, abduction of the arm is limited, and it is for this reason that many authors have condemned this technique (Jacobs and Wade, 1966: Quigley, 1963; Urist, 1963).

## Seventy‐five years ago

**O'Regan R. Perforating injuries of the rectum with case reports. *ANZ. J. Surg*. 1947; 16: 253–61.**

Injuries to the rectum by impalement are not common and their importance is not as widely recognized as it might be. Approximately 200 cases have been reported, but as far as I can find no complete survey of the literature has been made since 1912. The first extensive review of the subject was made by Van Hook, of Chicago, who reviewed the literature and collected 58 cases, adding two of his own. In 1900, Stiassny in a wide survey of the literature collected 127 cases. Tillman in 1905 added 16 more, and Habhegger in 1912 added another 36. This latter is the latest attempt at a complete compilation and it includes 175 cases. However, of this total there were only 70 cases in which the peritoneal cavity was involved. 'This is the group with which this article is concerned, and it is probable that less than 100 such cases have been recorded. Impalement of the rectum with perforation into the peritoneal cavity is a treacherous injury and the clinical picture may fail to reveal the extent and severity of the accident until all hope of successful treatment is past. Four cases of perforation of the rectum by impalement are described and their clinical features discussed. Attention is especially directed to the treacherous nature of injuries of this kind, because of the deceptive innocence of the clinical picture. These cases, together with similar cases from the available literature, are examined from the point of view of diagnosis, prognosis and treatment.

